# Supplementary material for: Prevention of acute rejection after rescue with Belatacept by association of low-dose Tacrolimus maintenance in medically complex kidney transplant recipients with early or late graft dysfunction
Source: PLoS One. 2020 Oct 15;15(10):e0240335. doi: 10.1371/journal.pone.0240335 (PMC7561183; doi:10.1371/journal.pone.0240335)
Supplement: Attachment — Submitted filename: Response to reviewers.docx [file pone.0240335.s002.docx]

*Editorial review*

*1. Please ensure that your manuscript meets PLOS ONE's style requirements, including those for file naming. The PLOS ONE style templates can be found at*

[*https://journals.plos.org/plosone/s/file?id=wjVg/PLOSOne_formatting_sample_main_body.pdf*](https://journals.plos.org/plosone/s/file?id=wjVg/PLOSOne_formatting_sample_main_body.pdf) *and*[*https://journals.plos.org/plosone/s/file?id=ba62/PLOSOne_formatting_sample_title_authors_affiliations.pdf*](https://journals.plos.org/plosone/s/file?id=ba62/PLOSOne_formatting_sample_title_authors_affiliations.pdf)

Response: We have modified our manuscript according to PLOS ONE's style requirements, including those for file naming.

*2. In ethics statement in the manuscript and in the online submission form, please provide additional information about the patient records used in your retrospective study. Specifically, please ensure that you have discussed whether all data were fully anonymized before you accessed them and/or whether the IRB or ethics committee waived the requirement for informed consent. If patients* *provided informed written consent to have data from their medical records used in research, please include this information.*

Response: Information about patients’ consent are now included in both Material and Methods section and Ethics Statement field of the submission form.

*3. Thank you for including your ethics statement:  "HUMAN RESEARCH.
The study was performed in adherence with the last version of the Helsinki Declaration and with the Principles of the Declaration of Istanbul on Organ Trafficking and
Transplant Tourism. All patients signed an informed consent before switching to Belatacept-based immunosuppressive therapy. This study is covered by Ethical
Committee approval, resolution number 1449/2019 on 11/08/2019 ("TGT" observational study)".*

*Please amend your current ethics statement to include the full name of the ethics committee/institutional review board(s) that approved your specific study.*

*Once you have amended this/these statement(s) in the Methods section of the manuscript, please add the same text to the “Ethics Statement” field of the submission form (via “Edit Submission”).*

Response: The full name of the ethics committee that approved our study is now included in both Material and Methods section and Ethics Statement field of the submission form.

*While revising your submission, please upload your figure files to the Preflight Analysis and Conversion Engine (PACE) digital diagnostic tool,* [*https://pacev2.apexcovantage.com/*](https://pacev2.apexcovantage.com/)*. PACE helps ensure that figures meet PLOS requirements. To use PACE, you must first register as a user. Registration is free. Then, login and navigate to the UPLOAD tab, where you will find detailed instructions on how to use the tool. If you encounter any issues or have any questions when using PACE, please email PLOS at* [*figures@plos.org*](mailto:figures@plos.org)*. Please note that Supporting Information files do not need this step.*

Response: We used Preflight Analysis and Conversion Engine (PACE) digital diagnostic tool for all our figures.

**Author response to reviewers’ comments**
*Reviewer #1: The tables are very clear and the test partition is very useful for understanding the study.*

*For personal interest, the autors can read a work of Vikas et al. (2014), that explain the aspects of kidney transplantation in children.*

Response: We really thank rev#1 for his/her comments, and we have included the suggested paper (*Vikas R. Dharnidharka et al, NEJM 2014*) in discussion *(Page 18, Line 296-298)*.

*Reviewer #2: The goal of this study, a suitable maintenance of immunosuppression regimen following kidney transplantations, clearly expressed in the title, is noteworthy. Nevertheless the important purpose of this work is hampered by methodological issues mainly featured by the choiche of the retrospective study method in face of a reduced sample size and without a real control group. Although the study results are consistent with the previous wider studies outcomes, these appear to be powerless. The authors are completely aware of this.
In this study relevant is the researchers interest to analyze medically complex patients otherwise excluded from the more powerfull Randomized Controlled Trials, but the worth efforts to manage highly complex patients deserve an higher patients number to provide more solid informations in this search field.
Minor issue concerns knowing more details about any other previous or concomitant immunosuppressant therapies in those patients received other organ transplants.
Finally it would be valued to obtain insights about metabolic data following belatacept therapy.*

Response: We appreciate Rev#2 considerations which allow us to improve the quality of our paper. Regarding the limits of our study, as mentioned, we are completely aware of all suggested limitations; however, we proposed some consideration about highly complex patients that, despite the (relative) low number included in our analysis, could be at high importance in this research area, and provided some information which may be useful for both clinicians and researchers.

Regarding immunosuppressive medications in patients with other organs, we added data about previous immunosuppressants in *Page 11, Line 180-182*.

Insights about metabolic data following belatacept therapy are now included in an appropriate section (Cardiovascular and methabolic changes, *Pages 16-17, Line 256-273*). Despite the absence of statistical significance, there was a trend in amelioration of some paramenters such as triglycerides. We deeply thank this reviewer to have oriented us to this issue since it can be a matter of further focused studies.
